# Supplementary material for: Emergence of high piezoelectricity from competing local polar order-disorder in relaxor ferroelectrics
Source: Nat Commun. 2023 Feb 23;14:1007. doi: 10.1038/s41467-023-36749-w (PMC9950361; doi:10.1038/s41467-023-36749-w)
Supplement: Supplementary file 1 — Supplementary Information [file 41467_2023_36749_MOESM1_ESM.pdf]

Supplementary Information

Emergence of high piezoelectricity from competing local polar order-disorder in relaxor ferroelectrics

Hui Liu[1,2,*,#], Xiaoming Shi[3,#], Yonghao Yao[1,2], Huajie Luo[1,2], Qiang Li[1], Houbing Huang[3,*], He Qi[1,2], Yuanpeng Zhang[4], Yang Ren[5], Shelly D. Kelly[6], Krystian Roleder[7], Joerg C. Neuefeind[4], Long-Qing Chen[8], Xianran Xing[1], Jun Chen[1,2*]

[1] Beijing Advanced Innovation Center for Materials Genome Engineering, University of Science and Technology Beijing, Beijing 100083, China

[2] Department of Physical Chemistry, University of Science and Technology Beijing, Beijing 100083, China

[3] School of Materials Science and Engineering, Beijing Institute of Technology, Beijing 100081, China

[4] Chemical and Engineering Materials Division, Oak Ridge National Laboratory, Oak Ridge, Tennessee 37831, USA

[5] Centre for Neutron Scattering, City University of Hong Kong, Kowloon, Hong Kong, China

[6] X-ray Science Division, Advanced Photon Source, Argonne National Laboratory, Argonne, IL 60439, USA

[7] Institute of Physics, University of Silesia, Katowice 40007, Poland

[8] Department of Materials Science and Engineering, Pennsylvania State University; University Park, PA 16802, USA

*Corresponding author. huiliu@ustb.edu.cn; hbhuang@bit.edu.cn; junchen@ustb.edu.cn

[#]These authors contributed equally: Hui Liu and Xiaoming Shi

Methods:

Sample Preparation. $Pb(Mg_{1/3}Nb_{2/3})O_3$-$x$$PbTiO_3$ ($20 \le x \le 45$) was prepared via solid-state route. The analytical reagent PbO, TiO_2, Nb_2O_5 and MgO powders were selected as raw materials. The homogeneous mixture powders were calcined at 850 ℃ for 4 h, and then sintered at 1225 ℃ for 2 h. The ceramics were crushed into fine powder and annealed at 400 ℃ for 2 h to relive the residual stresses for neutron and X-ray total scattering and Extended x-ray absorption fine structure (EXAFS) measurements.

Total Scattering Measurements. The neutron total scattering data were collected at room temperature at Nanoscale-Ordered Materials Diffractometer (NOMAD) at the Spallation Neutron Source (SNS), Oak Ridge National Laboratory. Approximately 1.5 g powder was placed in quartz capillary. The high quality data with Q_{max} about 35 $Å^{-1}$ were reduced using ADDIE software. The pair distribution function $G(r)$ were Fourier transformed from the corrected total scattering structure factors $S(Q)$. X-ray total scattering was measured at the 11-ID-C beamline of the Advanced Photon Source (APS, Argonne National Laboratory) using an incident-beam energy of ≈115 keV with the area detector positioned at 320 mm away from the sample. The sample was loaded in quartz capillary. The X-ray data were processed using the PDFGetX3 software,[1] to obtain the corresponding total-scattering function and its Fourier transform with Q_{max} = 22 $Å^{-1}$. The NIST Si SRM powder total scattering data were collected to identify the instrument resolution in both X-ray and neutron total scattering measurements. The calculated total-scattering data were corrected for the instrument resolution in both reciprocal and real spaces[2].

High Resolution Synchrotron X-ray Diffraction Measurements. The high resolution synchrotron X-ray diffraction were conducted at 11-BM-B beamline of APS with a wavelength of 0.45 Å. The powder samples were placed in a 0.3 mm diameter kapton capillary that was spun during the experiments. The diffractometer uses multiple single-crystal analyzer detectors to offer high-resolution ($\Delta Q/Q < 1.4\times10^{-4}$) data collection.

EXAFS experiments. The EXAFS of the Pb L_{III}-edge (13.035 keV) were performed at the 20-BM-B beamline at APS (Argonne National Laboratory). The X-ray absorption coefficient was measured in transmission mode as a function of the incident photon energy. The raw x-ray absorption spectrum processing and initial fitting were conducted on Athena and Artemis software[3,4]. Scattering amplitudes and phases were estimated

using FEFF8[5]. The k-space data were multiplied by k-weight of 1, 2 and 3 and the k-space range used in the Fourier transform was 2.1 Å^{-1} to 8-10 Å^{-1}, while the r-space fit was conducted from 1 Å to 3.5 Å, with single-scattering paths included. The data and fit range results in about 162 independent point each measurement. The Pb-O scattering paths were characterized by bond length determined from the big-box refinement. The high-quality Pb L_{III}-edge EXAFS data are typically difficult to collect. Considering the quality of EXAFS data, during the RMC fitting, the actual weight factor of Pb L_{III}-edge EXAFS data is smaller than the N-/X-PDF data.

Structural Analysis. The Bragg Rietveld refinement was done with GSAS-II software using the data from four banks (bank 2-bank 5). Reverse Monte Carlo (RMC) fitting was performed based on the RMCprofile software[6,7]. The 16×16×16 super-cell of pseudo-cubic structure (about 64 Å × 64 Å × 64 Å) with containing 20480 atoms and randomly distributed B site atoms were established based on the refined average unit cell. In the RMC simulations, the $G(r)$, $S(Q)$ and EXAFS data simultaneously were fitted under bond valence sum constraints and coordination constraints. B-site atoms were allowed to swap. Each RMC modelling was run to generate more than 10^3 moves per atom to get converged.

Calculation polar displacement and polar vectors. Based on the refined 3D atom configuration, the coordinates of the 12 neighboring oxygen atoms ($\vec{r}_{O_i}$) for each A site atoms ($\vec{r}_A$) can be extracted. The geometric center of the AO_{12} polyhedra can be calculated to be $\frac{1}{12}\sum_i \vec{r}_{O_i}$. The A site atom polar displacement vector ($\vec{D}_{A_i}$) can be calculated by the equation (1). Similarly, the surrounding six oxygen atoms ($\vec{r}_{O_i}$) of each B site atom ($\vec{r}_B$) can be found. The B site atom polar displacement vector ($\vec{D}_{B_i}$) can be calculated by the equation (2). Note that the A-site atom without 12 physically reasonable oxygen bonds, or these B-site atom without 6 physically reasonable oxygen bonds were discarded.

$$\vec{D}_{A_i} = \vec{r}_A - \frac{1}{12}\sum_i \vec{r}_{O_i} \tag{1}$$

$$\vec{D}_{B_i} = \vec{r}_B - \frac{1}{6}\sum_i \vec{r}_{O_i} \tag{2}$$

Calculation of polar vector ($\vec{P}$) in one perovskite unit cell. Firstly, the coordinates of 6 neighboring oxygen atoms ($\vec{r}_{O_i}$), and the coordinates of 8 neighboring *A* site atoms ($\vec{r}_{A_i}$) of each *B* site atom ($\vec{r}_{B_i}$) can be found. The 6 oxygen atoms, 8 A site atoms, and 1 *B*-site atom form one perovskite unit. In the perovskite unit, the center of the oxygen polyhedral is $\frac{1}{6}\sum_i \vec{r}_{O_i}$, and the center of the *A* site atom is $\frac{1}{8}\sum_i \vec{r}_{A_i}$. Subsequently, the polar vector ($\vec{P}$) can be calculated by considering a purely ionic crystal and neglecting the electronic polarization. According to the point charge model, the electric polarization $\vec{P}$ can be calculated by equation (3):

$$\vec{P} = \frac{q_A \times \left(\frac{1}{8}\sum_i \vec{r}_{A_i} - \frac{1}{6}\sum_i \vec{r}_{O_i}\right) + q_B \times \left(\vec{r}_{B_i} - \frac{1}{6}\sum_i \vec{r}_{O_i}\right)}{V} \quad (3)$$

To get the statistical analysis results about the polar displacement, four refined 3D atom configurations were merged together.

Phase-field simulations. Phase field modeling of PMN-PT are used to investigate the composition-dependent evolution of domain structures and piezoelectric response d_{33}. The domain structures are described by the spatial distribution of spontaneous polarization $\boldsymbol{P}$ (P_1, P_2, P_3). The temporal evolution of the polarization is described by the time-dependent Ginzburg-Landau (TDGL) equation, the displacement field $\boldsymbol{u}$ and electric displacement field *D* are solved with the stress/electric equilibrium equation[8],

$$\begin{aligned} &\frac{\partial P_i}{\partial t} = -L\frac{\delta F}{\delta P_i} + E_i^{thermal} \\ &\frac{\partial}{\partial x_j}(\sigma_{ij}(r,t)) = 0 \\ &\nabla \cdot D = \rho_f \end{aligned} \quad (4)$$

Here, *L* is a kinetic coefficient related to domain wall mobility, *F* is the total free energy of the system, $\frac{\delta F}{\delta P_j}$ is the thermodynamic driving force for polarization evolution, σ_{ij} is the stress tensor , ρ_f is the free charge density. *r* and *t* are the spatial coordinate and time, respectively. The total free energy of a bulk system can be defined as follows,

$$F = F_{bulk}(\boldsymbol{P}) + F_{grad}(\boldsymbol{P}) + F_{elastic}(\boldsymbol{P}) + F_{elec}(\boldsymbol{P},\boldsymbol{E}) = \int_V (f_{bulk} + f_{grad} + f_{elastic} + f_{elec})dV \quad (5)$$

Here, F includes the bulk free energy $F_{bulk}(\boldsymbol{P})$, domain-wall energy $F_{grad}(\boldsymbol{P})$, elastic energy $F_{elastic}(\boldsymbol{P})$, and electrostatic energy $F_{elec}(\boldsymbol{P}, \boldsymbol{E})$, where E is the applied static electric field. f_{Land}, f_{grad}, $f_{elastic}$ and f_{elec} are corresponding energy densities.

The bulk free-energy density is expressed for zero strain as a six-order polynomial expansion, that is

$f_{bulk} = \alpha_1(P_1^2 + P_2^2 + P_3^2) + \alpha_{11}(P_1^4 + P_2^4 + P_3^4) + \alpha_{12}(P_1^2P_2^2 + P_2^2P_3^2 + P_1^2P_3^2) + \alpha_{111}(P_1^6 + P_2^6 + P_3^6) + \alpha_{112}[P_1^4(P_2^2 + P_3^2) + P_2^4(P_1^2 + P_3^2) + P_3^4(P_1^2 + P_2^2)] + \alpha_{123}P_1^2P_2^2P_3^2$ (5)

where α_i, α_{ij}, α_{ijk} are the Landau parameters. The elastic energy density can be written as $f_{elas} = \frac{1}{2}C_{ijkl}(\varepsilon_{ij}-\varepsilon_{ij}^0)(\varepsilon_{kl}-\varepsilon_{kl}^0)$, where C_{ijkl} is the elastic stiffness tensor, ε_{ij} is the total strain and ε_{ij}^0 is the eigenstrain. The eigenstrain can be described as $\varepsilon_{ij}^0 = Q_{ijkl}P_kP_l$, where Q_{ijkl} is the electrostrictive coefficient. The gradient energy density can be obtained by $f_{grad} = \frac{1}{2}G_{ijkl}\, P_{i,j}\, P_{k,l}$, where G_{ijkl} is the gradient coefficient.

The electrostatic energy can be expressed as $f_{elec} = -\frac{1}{2}(\boldsymbol{E}\cdot\boldsymbol{P})$, where $\boldsymbol{E}$ is the total electric field, which can be described as $E = E_{appl} + E_{dipole} + E_{RF}$, where E_{appl} is the applied electric field, E_{dipole} is the dipole-dipole interaction field, and E_{RF} is the local electric field caused by the random point defects.

The simulation scale is 256dx×256dz, the grid scales dx and dz are 1 nm. The Fourier method was used for solving the equations. The parameters in detail are selected from Ref.9. Take PMN-0.3PT as an example, the parameters are, T_C = 408K, $\alpha_1 = (2.2295T-935.9)\times10^5$ m/F, $\alpha_{11} = (-0.3775T+457.7)\times10^5$ m^5/C^{2}F, $\alpha_{12} = 6.075\times10^7$ m^5/C^{2}F, $\alpha_{111} = 2.57\times10^9$ m^9/C^{4}F, $\alpha_{112} = 6.95\times10^9$ m^8/C^{4}F, $\alpha_{113} = 13.13\times10^9$ m^8/C^{4}F, Q_{11} = 0.084 m^4/C^2, Q_{12} = –0.0025 m^4/C^2, Q_{44} = 0.035 m^4/C^2, $s_{11} = 52\times10^{-12}$ m^2/N, $s_{12} = -18.9\times10^{-12}$ m^2/N, $s_{44} = 14\times10^{-12}$ m^2/N. The gradient energy coefficients are chosen to be $G_{11}/G_{10} = 1.5$, $G_{12}/G_{110} = 0.0$, $G_{44}/G_{110} = 0.75$, where $G_{110} = 7.04\times10^{-11}$ C^{-2}m^4/N. The Landau coefficients can produce a transition from R phase to T phase with the composition change.

Here, a random electric field that obeys the Gaussian distribution $N(0, \Delta)$ was applied to account for the E_{RF}, where Δ is the variance of the Gaussian distribution. Based on the random field theory for relaxors, local random fields are introduced to characterize the effect of chemical disorder. With PT doping from x=0.2 to x=0.45, the

random local electric field variance decreased from 30 kV/mm to zero. During modeling, two effects are employed. Firstly, composition-induced MPB will lower the energy barrier compared with the single R/T phase, which is supposed to lead to a maximum d_{33} value in MPB. However, the calculated d_{33} from phase-field method will not decrease with decreased x value ($x < 0.33$), which may originate from the low energy barrier in the R phase. Secondly, the increased randomness of polar order parameters is characterized by an increase in random local electric field variance. With the random field effect, the calculated d_{33} decreased to ~233 pC/N ($x = 0.2$) and had a maximum value ($d_{33} = 753$ pC/N) in MPB ($x = 0.33$), consistent with the experiment result. On the other hand, the introuding of local random fields makes the final domain structures more accordance with actual observed ones.

Supplementary Figures

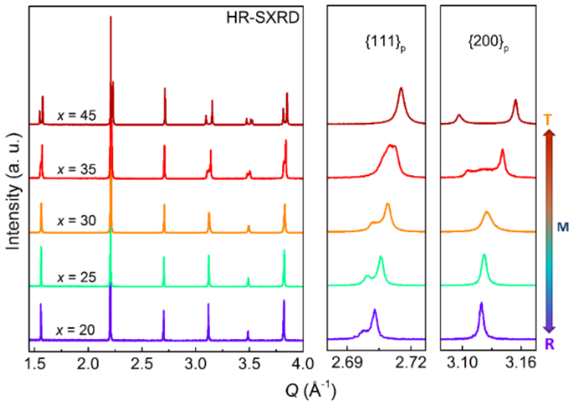

Supplementary Fig. 1. Long-range average crystal structre evolution. High-resolution synchrotron X-ray powder diffraction patterns of PMN-PT.

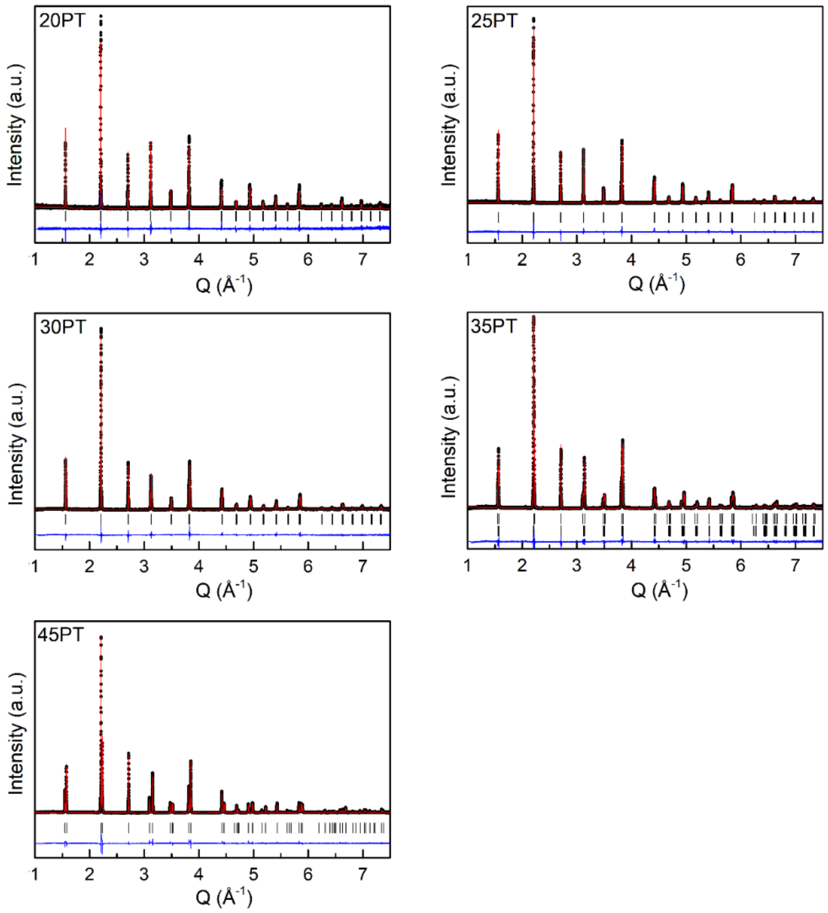

Supplementary Fig. 2. Long-range average crystal structre evolution. The Rietveld refinement results agaisnt high-resolution synchrotron X-ray powder diffraction patterns of PMN-PT. The observed data (black dots), the calculated profile (red line), and the difference between the observed and calculated patterns (bottom bule line) are depicted. The thick marks indicate the Bragg peak positions.

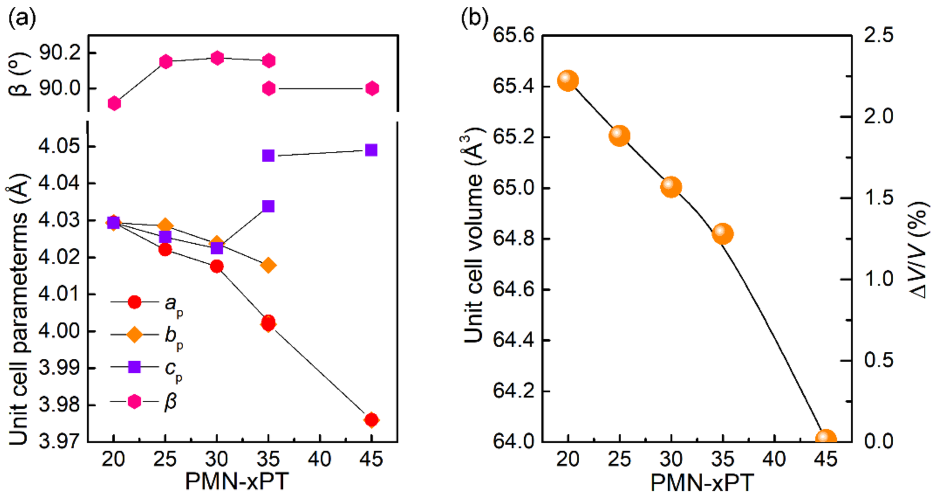

Supplementary Fig. 3. Long-range average crystal structre evolution. Unit cell parameters in pseudo-cubic unit set of PMN-*x*PT obtained from the Rietveld refinement against the high-resolution synchrotron X-ray powder diffraction patterns. 20PT: *R*3*m*, 25PT: M_B (*Cm*), 30PT: M_A (*Cm*), 35PT (31% *P*4*mm*, and 69% *Pm*), and 45PT: *P*4*mm*. (a) Unit cell parameters and (b) The unit cell volume and relative variation of unit cell volume with respect to 45PT. With increasing PMN contnent the unit cell volume expands, which is necessary to introduce randomness in solid solutions. Futher, this evolution is consistent with the revealed incresing disorder of polar vectors.

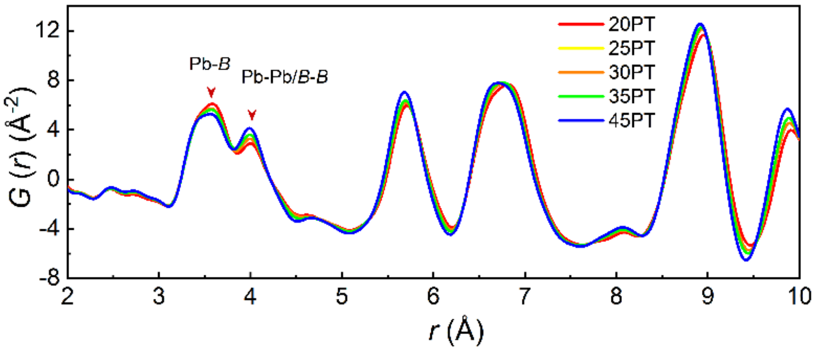

Supplementary Fig. 4. Local structre evolution. X-ray atomic pair distribution functions (PDFs) $G(r)$, determined from X-ray total scattering in the interatomic distance r range of 1–10 Å, showing the evolution of short-range atom-atom correlations as a function of composition in PMN-*x*PT. The abscissa represents the interatomic distance, and ordinate represent the intensity of the reduced pair distribution function $G(r)$.

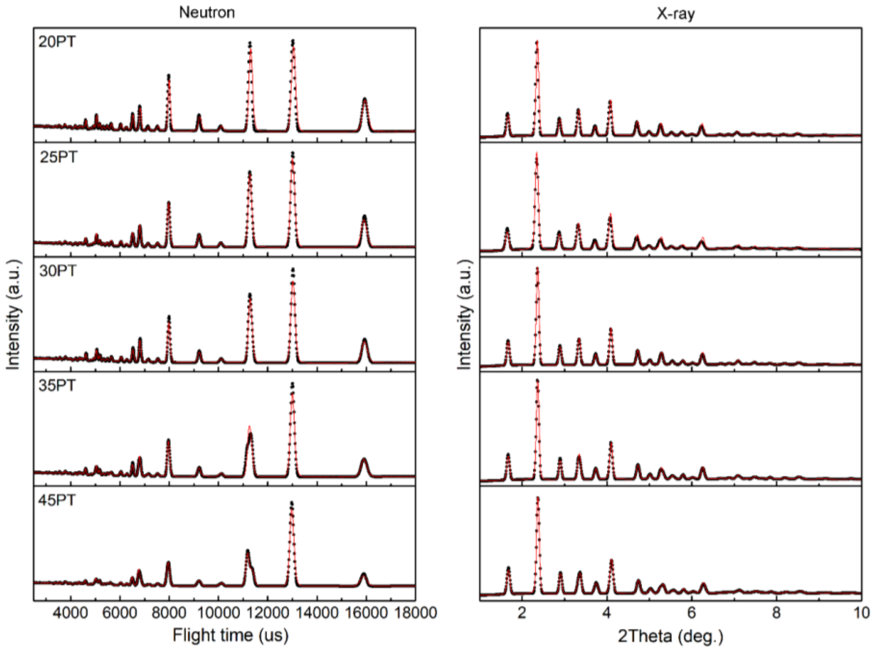

Supplementary Fig. 5. Results of RMC fitting. Fitted neutron time-of-flight and X-ray Bragg data using RMC method in comparison with experimental data at room temperature of PMN-PT. The black points and red line indicates the observed and calculated data, respectively.

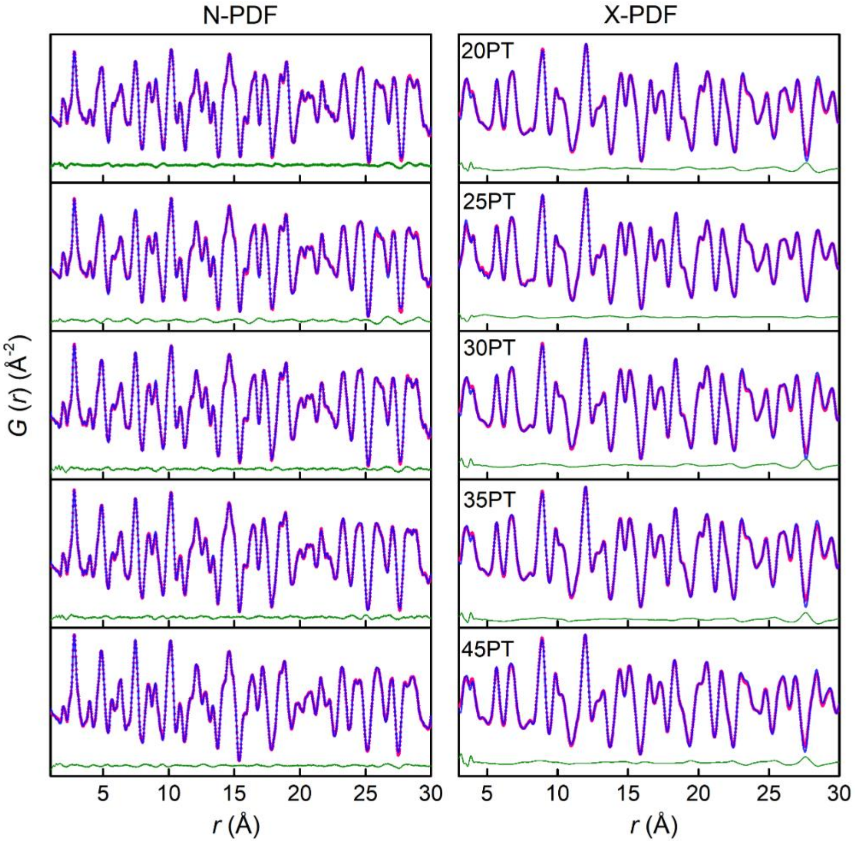

Supplementary Fig. 6. Results of RMC fitting. Fitted and observed neutron and X-ray PDF *G*(*r*) of PMN-PT. The pink point, blue line and green line indicates the observed data, RMC calculated data, and difference, respectively. Both neutron and X-ray PDF *G*(*r*) were corrected for the instrument resolution by using the NIST Si standard.

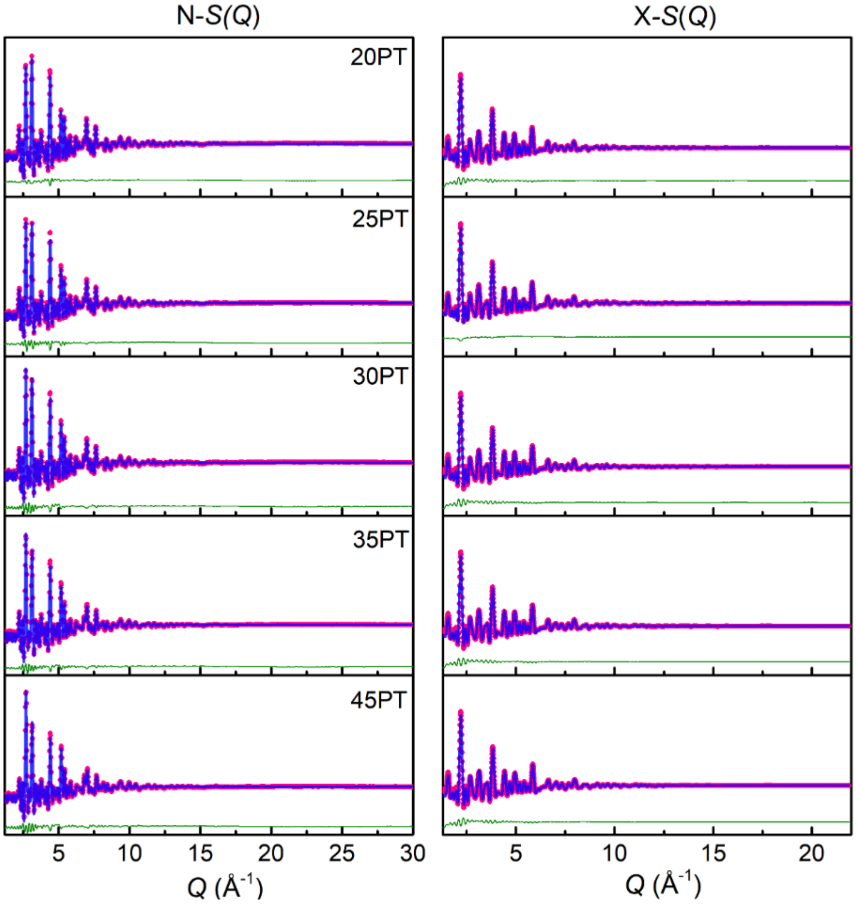

Supplementary Fig. 7. Results of RMC fitting. Fitted and observed neutron and X-ray *S*(*Q*) of PMN-PT. The pink point, blue line and green line indicates the observed data, RMC calculated data, and difference, respectively. Both neutron and X-ray *S*(*Q*) were corrected for the instrument resolution by using the NIST Si standard.

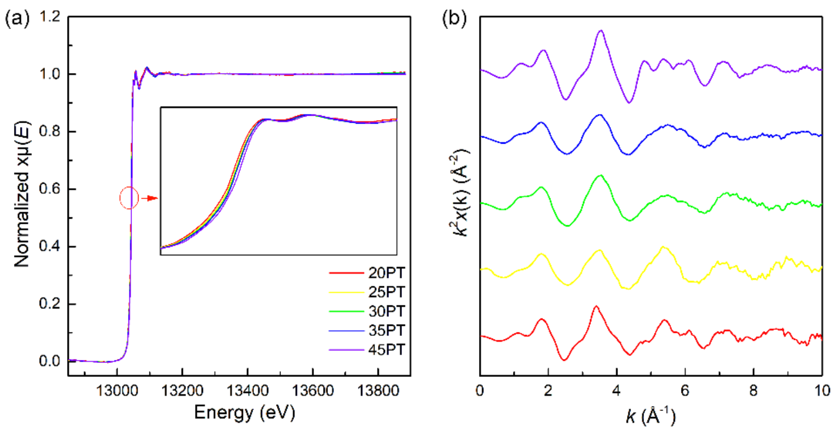

Supplementary Fig. 8. Measured EXAFS data. (a) Normalized Pb L_{III}-edge X-ray absorption near edge structure spectra of PMN-*x*PT. The inset shows the enlarged spectra at absorption edge. (b) The Pb L_{III}-edge XANES spectra of *k*-weighted form of $k^2\chi(k)$ as a function of wavenumber *k* of PMN-*x*PT. The high-quality Pb L_{III}-edge EXAFS data are typically difficult to collect. The quality of EXAFS data in present study are comparable with previous reported in the literatures[10,11]. Considering the quality of EXAFS data, during the RMC fitting, the actual weight factor of Pb L_{III}-edge EXAFS data is smaller than the neutron and X-ray PDF data.

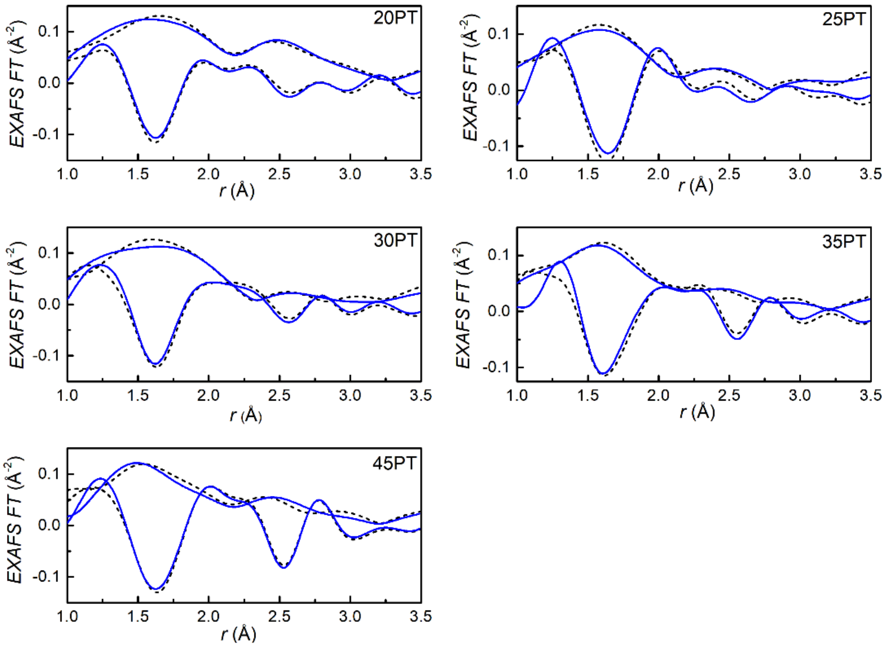

Supplementary Fig. 9. Results of RMC fitting. Pb EXAFS fitting of PMN-PT. The blue line and black dotted line indicates the observed and RMC calculated data, respectively. The magnitude and imaginary part (dashed line) of the Fourier transform are shown.

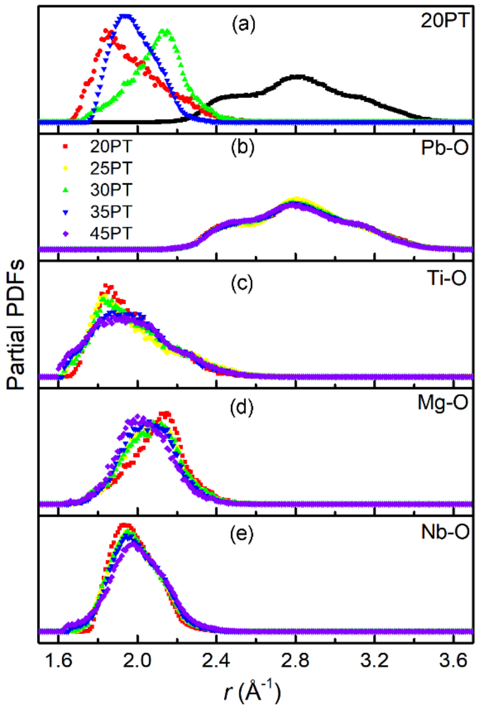

Supplementary Fig. 10. Results of RMC fitting. Element specific partial *A/B*–O PDFs obtained from the refined 3D atomic configuration. (a) Representative partial *A/B*–O PDFs of 20PT. The compositional dependent partial *A/B*–O PDFs, (b) Pb-O, (c) Ti-O, (d) Mg-O, (e) Nb-O.

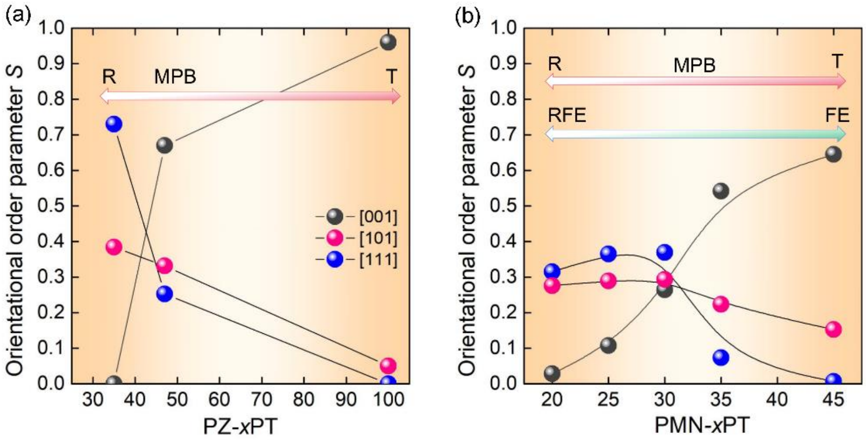

Supplementary Fig. 11. Comparison of composition-dependent orientation order parameter *S*. (a) the conventional ferroelectric system of PZT, and (b) the relaxor ferroelectric system of PMN-PT. The order parameter *S* of conventional Pb(Zr,Ti)O_3 (PZT) ferroelectric system, including the *T* composition of $PbTiO_3$ (PT), *R* composition of $PbZr_{0.65}Ti_{0.35}O_3$ (PZT65) and MPB composition of $PbZr_{0.53}Ti_{0.47}O_3$ (PZT53) were extracted from RMC refined 3D atomic configuations. The MPB composition of PZT53

presents multiphase coexistence, but do not have relaxor feature. For PZT system, the orientational order parameter *S* of local polar vectors along [001] direction decreases, accompanied by the increasing in *S* along [111] direction from *T*-to-MPB-to-*R*. For the PMN-PT relaxor system, with composition from *T* approaching MPB, the orientational order parameter *S* along [001] direction reduced, and increase along [111] direction. While the order parameter *S* along three directions ([001], [110], and [111]) deceases from MPB-to-*R*, which is completely different with the PZT system. This arises from the relaxor feature. Specifically, for MPB composition PZT53, the polar disorder is from the multiphase coexistence, and leads to a less polar disorder (disorder parameter ξ = 0.25). In PMN-PT system, the polar disorder is not only from the multiphase coexistence, but also from the existence of relaxor feature (disorder parameter ξ = 0.5 in 30PT). The existence of relaxor feature leads to higher degree disorder polar direction in PMN-30PT compared with conventional PZT53. Macroscopically, the PMN-PT system displays much higher piezoelectric response ($d_{33} \approx 700$ pC/N) compared with PZT system ($d_{33} \approx 220$ pC/N). Therefore, the mechanism of competing of local polar order-disorder is beyond the multiphase coexistence. The multiphase coexistence cannot rationalize the scenario that relaxor systems present several times higher piezoelectric coefficients compared to classical ferroelectric counterparts.

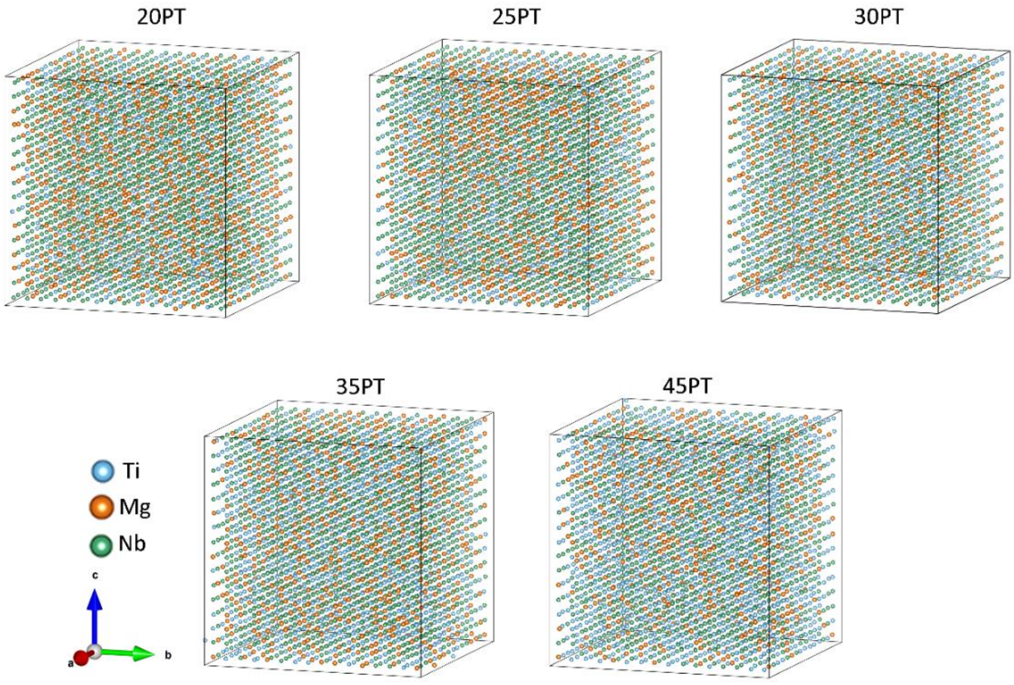

Supplementary Fig. 12. The distributions of *B* site atoms. The distributions of *B* site

atoms in one of refined configurations of PMN-*x*PT.

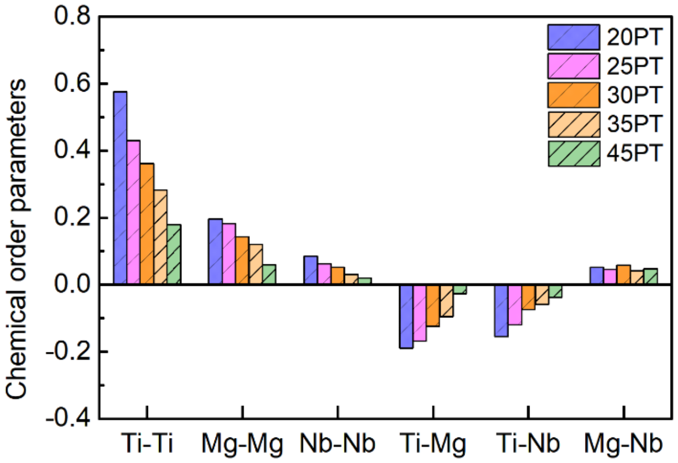

Supplementary Fig. 13. Chemical order of PMN-PT. The Warren-Cowley parameters of first shell neighbor correlations for the *B* site atoms of PMN-PT. The chemical order parameters (so-called Warren-Cowley parameters), were calculated from the refined atomic configuration to evaluate the *B*-site chemical order in the PMN-PT. The calculated the chemical order parameter in the 1st shell of *B*-site atom. Note that positive values indicate ordered structure preference of like atoms as nearest neighbors, and negative ordered structure preference of unlike atoms as nearest neighbors.

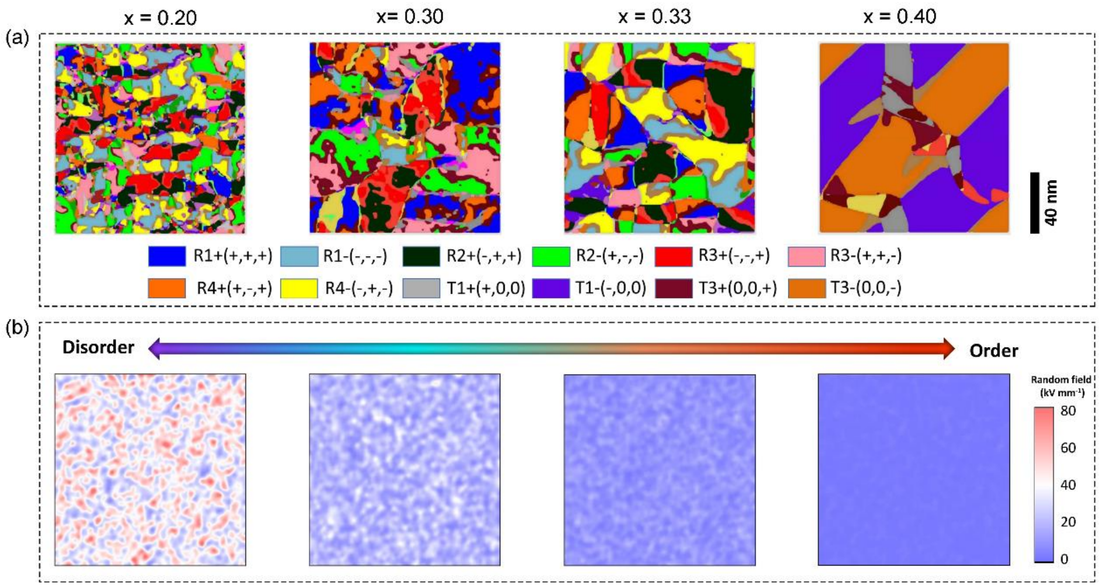

Supplementary Fig. 14. Polar structures simulated by the phase-field simulation. (a) Domain structures calculated from phase-field simulation. The main domain

structures are represented by different colors. (b) The local random field distribution. The effect of polar disorder is characterized by the local random field variance. A larger random field corresponds to higher degree of polar disorder. The composition-dependent local electric field variance with amplitude positively related to the volume fraction of polar-disorder regions was introduced. Correspondingly, the random field variance decreased from 30 kV/mm in 20PT to 0 kV/mm in 45PT. The domain size decreases, and the domain structures change obviously from 40PT to 20PT.

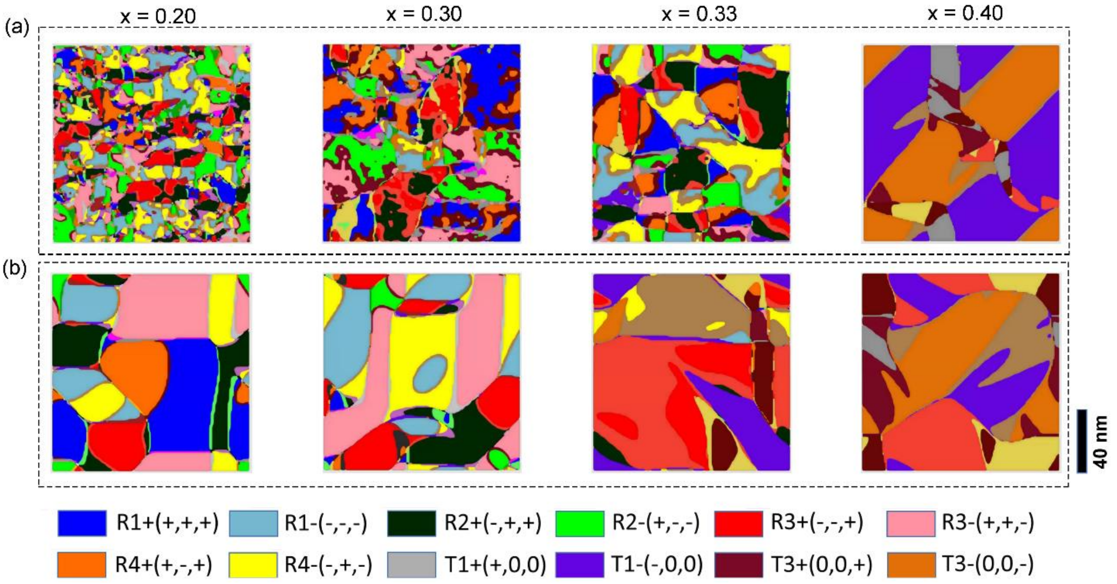

Supplementary Fig. 15. Polar structures simulated by the phase-field simulation. Cmaprsion of the domain structures calculated from phase-field simulation between with (a) and without (b) introducing random electric fields. In the phase-field simulations, random electric field are commonly used to simulate the relaxor behavior in relaxors due to the chemcial disorder[12,13]. Without introducing random electric field, one can not get the correct Landau energy profiles, and thus unable to get the correct domain strcutres and piezoelctirc response. One can clearly see that the domain structures are completely different between these with introduction of random electric field (consider as disorder) and these without. The local polar direction randomness (random electric field) can break the long-range ferroelectric order. Thus, the introduction of random electric field reduces the domain size and make the domain wall dispersion, these important polar characteristic are more consistent with what's actually observed.

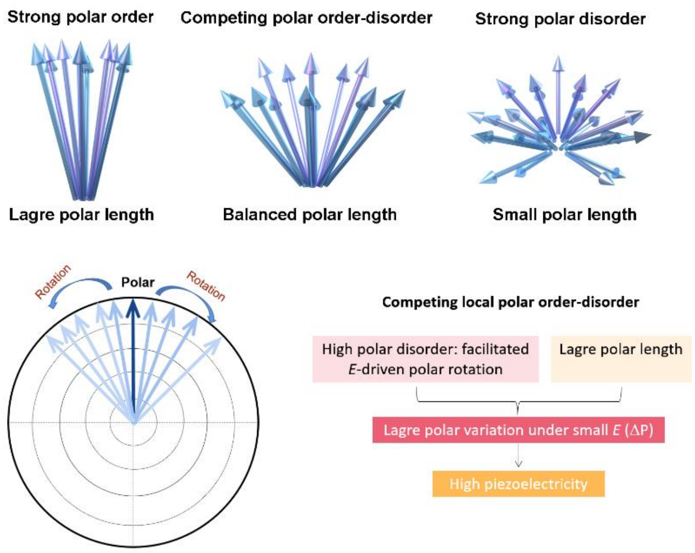

Supplementary Fig. 16. Schematic diagram of high piezoelectricity. Schematic diagram of high piezoelectricity emerges from the competing of the local polar order-disorder between different states and the balanced local polar length and direction randomness.

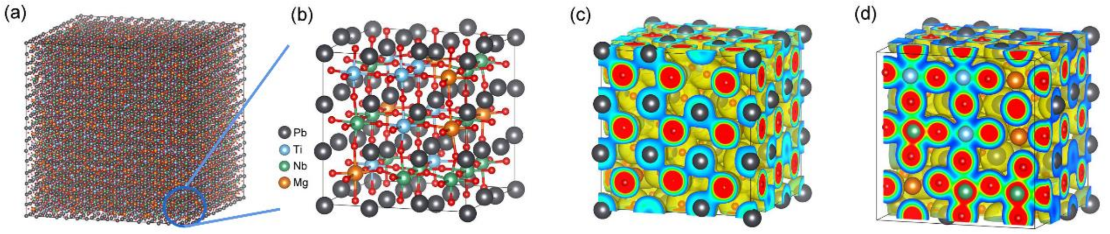

Supplementary Fig. 17. Charge density distribution. (a) One of the RMC refined 3D atomic configurations of PMN-35PT. (b) Selected atomic configurations for calculating charge density distribution. The charge density distribution in Pb-O plane (c), and *B*-O plane (d). The strong hybridization between Pb 6*s* with O 2*p* states occurs in every perovskite unit-cell. The Mg-O bonds are ionic type, no hybridization between Mg and O is observed. Covalent bonds of Ti/Nb-O are observed due to the hybridization of their *4d* with O *2p* states. These results demonstrate that the local polarization is stabilized mainly by the strong hybridization between Pb 6*s* with O 2*p* states. Besides, the hybridization of Ti/Nb *4d* with O *2p* states plays some part.

Supplementary References

1 P. Juhas, T. Davis, C. L. Farrow, S. J. L. Billinge, PDFgetX3: A rapid and highly automatable program for processing powder diffraction data into total scattering pair

distribution functions. *J. Appl. Cryst.* **46**, 560-566 (2013).

2 Zhang, Y. et al. New capabilities for enhancement of RMCProfile: instrumental profiles with arbitrary peak shapes for structural refinements using the reverse Monte Carlo method. *J. Appl. Cryst.* **53**, 1509-1518 (2020).

3 Ravel, B. & Newville, M. ATHENA, ARTEMIS, HEPAESTUS: data analysis for X-ray absorption spectroscopy using IFEFFIT. *J. Synch. Rad.* 12, 537–541 (2005).

4 Swartz, S. L. & Shrout, T. R. Fabrication of perovskite lead magnesium niobate. *Mat. Res. Bull.* **17**, 1245–1250 (1982).

5 A. L. Ankudinov, B. Ravel, J. J. Rehr, S. D. Conradson, Real-space multiplescattering calculation and interpretation of X-ray absorption near-edge structure. *Phys. Rev. B* **58**, 7565–7576 (1998).

6 M. G. Tucker, D. A. Keen, M. T. Dove, A. L. Goodwin, Q. Hui, RMCProfile: reverse Monte Carlo for polycrystalline materials. *J. Phys. Cond. Matter* **19**, 335218 (2007).

7 V. Krayzman, I. Levin, J. C. Woicik, T. Proffen, T. A. Vanderah, M. G. Tucker, A combined fit of total scattering and extended Xray absorption fine structure data for local-structure determination in crystalline materials. *J. Appl. Crystallogr.* **42**, 867-877 (2009).

8 Y. L. Li, S. Y. Hu, L. Q. Chen, Ferroelectric domain morphologies of (001) $PbZr_{1-x}Ti_xO_3$ epitaxial thin films. *J. Appl. Phys.* **97**, 1 (2005).

9 A. A. Heitmann, G. A. Rossetti, Thermodynamics of ferroelectric solid solutions with morphotropic phase boundaries. *J. Am. Ceram. Soc.* **97**, 1661 (2014).

10 Yoneda, Y. et al. Local structure analysis of relaxor $Pb(Mg_{1/3}Nb_{2/3})O_3$. *Ferroelectrics* **513**, 1 (2017).

11 Eremenko, M. et al. Local atomic order and hierarchical polar nanoregions in a classical relaxor ferroelectric. *Nat. Commun.* **10**, 2728 (2019).

12 Wang, S. et al. A phase-field model of relaxor ferroelectrics based on random field theory, *Int. J. Solids Struct.* **83**, 142–153 (2016).

13 Hong, Z. et al. Role of point defects in the formation of relaxor ferroelectrics. *Acta Mater.* **225,** 117558 (2022).
